# Properties of *Ginkgo biloba* L.: Antioxidant Characterization, Antimicrobial Activities, and Genomic MicroRNA Based Marker Fingerprints

**DOI:** 10.3390/ijms21093087

**Published:** 2020-04-27

**Authors:** Katarína Ražná, Zuzanna Sawinska, Eva Ivanišová, Nenad Vukovic, Margarita Terentjeva, Michal Stričík, Przemysław Łukasz Kowalczewski, Lucia Hlavačková, Katarína Rovná, Jana Žiarovská, Miroslava Kačániová

**Affiliations:** 1 Department of Plant Genetics and Breeding, Faculty of Agrobiology and Food Resources, Slovak University of Agriculture, Tr. A. Hlinku 2, 949 76 Nitra, Slovakia; katarina.razna@uniag.sk (K.R.); lucia.hlavackova1@gmail.com (L.H.); jana.ziarovska@uniag.sk (J.Ž.); 2 Department of Agronomy, Poznań University of Life Sciences, 11 Dojazd St., 60-632 Poznań, Poland; zuzanna.sawinska@up.poznan.pl; 3 Department of Technology and Quality of Plant Products, Faculty of Biotechnology and Food Sciences, Slovak University of Agriculture, Tr. A. Hlinku 2, SK-94976 Nitra, Slovakia; eva.ivanisova@uniag.sk; 4 Department of Chemistry, Faculty of Science, University of Kragujevac, P.O. Box 12, 34000 Kragujevac, Serbia; nvchem@yahoo.com; 5 Institute of Food and Environmental Hygiene, Faculty of Veterinary Medicine, Latvia University of Life Sciences and Technologies, K. Helmaņaiela 8, LV-3004 Jelgava, Latvia; margarita.terentjeva@llu.lv; 6 Department of Economy, The Faculty of Business Economics, University of Economics in Bratislava, 852 35 Košice, Slovakia; michal.stricik@euke.sk; 7 Institute of Food Technology of Plant Origin, Poznań University of Life Sciences, 31 Wojska Polskiego St., 60-624 Poznań, Poland; przemyslaw.kowalczewski@up.poznan.pl; 8 Department of Planting Design and Maintenance, Faculty of Horticulture and Landscape Engineering, Slovak University of Agriculture, Tr. A. Hlinku 2, 94976 Nitra, Slovakia; katarina.rovna@uniag.sk; 9 Department of Fruit Sciences, Viticulture and Enology, Faculty of Horticulture and Landscape Engineering, Slovak University of Agriculture, Tr. A. Hlinku 2, 94976 Nitra, Slovakia; 10 Department of Bioenergy, Food Technology and Microbiology, Institute of Food Technology and Nutrition, University of Rzeszow, 4 Zelwerowicza St., 35601 Rzeszow, Poland

**Keywords:** *Ginkgo biloba*, antimicrobial activity, polyphenols, flavonoids, antioxidant, microRNA, miRNA-based markers

## Abstract

The aim of this study was to characterize extracts from the leaves of *Ginkgo biloba* L. from selected Slovakian localities in terms of the content of bioactive constituents, antioxidants and their antimicrobial properties. The results indicated that the content of antioxidants was sample-specific, and this specificity was statistically significant. *Ginkgo biloba* L. from the locality of Košice had the best activity determined by the free radical scavenging activity (DPPH) (1.545 mg Trolox equivalent antioxidant capacity (TEAC)/g fresh matter (FM)) as well as the molybdenum-reducing antioxidant power (35.485 mg TEAC/g FM) methods. The highest content of total polyphenols (2.803 mg gallic acid equivalent (GAE)/g FM) and flavonoids (4.649 μg quercetin equivalent (QE)/g FM) was also detected in this sample. All samples of *G. biloba* leaf extracts showed significant antimicrobial activity against one or more of the examined bacterial species, and *Staphylococcus aureus* subsp. aureus CCM 2461 was found to be the most susceptible (minimal inhibition concentration MIC50 and MIC90 values of 64.2 and 72.2 µg/mL, respectively). Based on the results it was concluded that *Ginkgo biloba* L. extracts can be used as antimicrobial and antioxidant additives. Selected miRNA-based molecular markers were used to examine the environmental adaptability of *Ginkgo biloba* L. An almost-complete genotype clustering pattern based on locality was determined in the analysis that involved a species-specific gb-miR5261 marker. Morphologically specific exemplar, cv. Ohatsuki, was excluded.

## 1. Introduction

Many plants are well known for a diverse range of bioactive molecules, making them a rich source of different types of drugs. Most of the medicines used today are acquired from natural products that have been used in traditional medicine [1,2,3]. The use of plant extracts and phytochemicals, well known for their antioxidant and antimicrobial properties, can be of a great importance in therapeutic treatments. A major part of the population of developing countries still uses traditional medicine based on the significant antioxidant and antimicrobial properties of plants [4,5]. About 80% of individuals from well-developed countries use traditional therapeutic treatments that are based on compounds obtained from medical plants. Recently, a number of studies in various countries around the world have determined antioxidant and antimicrobial effects of a wide range of plants, and proved their efficiency. Antioxidant and antimicrobial agents are a result of the secondary metabolism of plants [6] which, therefore, should be investigated in order to better understand their properties, safety and efficacy [7].

*Ginkgo biloba* L. is considered a living fossil as it has survived over millions of years [8]. It is one of the oldest living tree species. *Ginkgo* survived without structural modifications for over 200 million years. It contains a number of biologically active compounds that act as defense measures against insects, bacteria and fungi. A number of secondary metabolites, such as terpenoids, polyphenols, organic acids and amino acids, have been isolated from the plant [9]. *Ginkgo biloba—*especially its leaves*—*are rich sources of compounds with antioxidant activity. The main compounds responsible for this kind of activity were identified as kaempferol, quercetin and isorhamnetin. These flavonoids can scavenge and destroy free radicals, especially peroxide, hydrogen peroxide, hydroxyl radical and singlet oxygen, which are connected with many diseases such as carcinogenesis, inflammation, atherogenesis, as well as food deterioration. For this reason the naturally occurring antioxidants in *Ginkg*o may be effective for different medical applications [10,11]. *Ginkgo* extracts are known for their antiparasitic, antifungal, antioxidant, antibacterial, antiviral, and DNA protective activities [12,13,14]. In China, *Ginkgo* extracts have been used for 5000 years to treat lung infectious diseases such as bronchitis and also as a remedy for cardiovascular diseases [15]. Extracts from the leaves of *Ginkgo* have been used in Chinese medicine from ancient times. In Western countries such as Germany and France, leaf extracts of *Ginkgo biloba* L. have been used since the 1960s for treatment of atherosclerosis and cerebrovascular insufficiency [16,17]. It has also been used to treat depression, memory loss, headaches and vertigo because of its positive effects on circulation [18].

Molecules of microRNA (miRNA) are highly conserved and hence can be useful for studying genetic diversity among closely related genotypes [19] and related species [20]. These novel markers of functional potential have been applied only to a limited extent in plant species. MicroRNA marker assays have been performed in monocotyledonous species as foxtail millet [20], rice [19,21] and dicotyledonous *Brassica* species [22]. So far, no reports have focused on genotyping of the gymnosperm *Ginkgo biloba* as a plant with an interesting genetic background of environmental adaptability and medicinal importance.

The aim of this study was to investigate the chemical composition, antioxidant and antimicrobial activity of ethanolic leaf extract of *Ginkgo biloba* L. from selected localities in Slovakia using in vitro methods. Our results show new information about antimicrobial activity and characterization of genotypes. Moreover, the potential of different types of microRNA molecular markers as a tool of resolution and characterization of ginkgo genotypes with respect to microecological conditions and the quantified parameters was also evaluated.

## 2. Results and Discussion

### 2.1. Quantitative Analysis of G. biloba Leaf Extracts

The results of quantitative analysis obtained by LC-MS/MS in data-dependent multiple reaction monitoring (MRM) mode were given in Table 1.

As can be seen in Table 1, the most abundant compound present in all the extracts was quercetin-3-O-rutinoside (211.23 μg/g to 498.38 μg/g). Chlorogenic acid and quercetin were the dominant phenolics in sample S2 (223.35 μg/g and 223.99 μg/g, respectively). Other chemotaxonomic markers of *G. biloba* leaf extracts, such as isorhamnetin, luteolin, kaempferol and their glycosides were observed in small amounts. The exception is luteolin, which was abundant in sample S8 (111.23 μg/g).

### 2.2. Antioxidant Activity

While all the extracts showed antioxidant activity (Table 2), the best results were noted for samples from the localities of Košice (1.545 mg Trolox equivalent antioxidant capacity (TEAC)/g fresh matter (FM)) and Palárikovo (1.338 mg TEAC/g FM). Strong activity was also detected in samples from the localities of Lučenec (1.333 mg TEAC/g FM) and Budimír (1.224 mg TEAC/g FM). Our results showed that agro-ecological conditions can influence antioxidant activity. Several parameters, such as weather, soil, varieties, stages of maturity and climatic conditions during the year, influence the amount of bioactive compounds in medicinal herbs [23,24]. Kobus et al. [25] studied antiradical activity of water, ethanolic and aqueous acetonic extracts from *Ginkgo* green and yellow leaves. Aqueous acetonic (4.89 mM TEAC/g dry matter) and ethanolic extracts (1.02 mM TEAC/g) from green leaves showed a greater degree of eliminated DPPH radical than extracts from yellow *Ginkgo* leaves. In their study a very interesting result was that water infusion extract from yellow leaves (2.91 mM TEAC/g) had better activity than extract from green leaves (2.22 mM TEAC/g). Many studies confirmed that the scavenging of DPPH radicals is connected from the spectrum of individual chemical components. The antiradical properties of *Ginkgo* extracts, for which the flavonoid fraction is probably responsible, can eliminate radicals such as the reactive oxygen species and reactive sulfur species. The content of individual types of biologically active compounds are closely related to their accumulation levels over the course of the growing season; during this time, both the proportions and forms of the bioactive compounds vary [25,26,27].

The molybdenum reducing antioxidant power is based on the reduction of Mo(VI) to Mo(V) by the extract and subsequent formation of green phosphate/Mo(V) complex at acid pH. This method evaluates both water-soluble and fat-soluble antioxidants (total antioxidant capacity). The results indicate (Table 2) that the highest reducing power was found in the sample from the locality of Košice (35.485 mg TEAC/g FM) followed by samples from Lučenec (32.545 mg TEAC/g FM) and Šurianky (26.835 mg TEAC/g FM) localities. It was noticed that the electron-donating capacity, reflecting the reducing power of bioactive components, is associated with antioxidant activity. Antioxidants act as reductants, and inactivation of oxidants by reductants can be described as redox reactions in which one reaction species is reduced at the expense of the oxidation of the other [28]. In the study of Kobus et al. [25], the highest absorbance values, were determined in aqueous acetone extract of yellow leaves which was followed by the water infusion from yellow leaves. Stefanovits-Banyai et al. [29] tested the reducing power of extracts prepared from *Ginkgo* leaves. These authors described several factors which can influence biological activity; they compared the reducing power of water infusion and ethanolic extracts from leaves of individual *Ginkgo* samples. The aqueous extracts reduced iron to a lesser degree than the ethanolic extracts. In their studies, however, the authors used 80% (v/v) ethanol solutions, which—as noted by the authors—extract more polyphenols. It is the polyphenols that are probably mainly responsible for reducing power compared with the water infusion preparations. In our study, 80% (*v*/*v*) ethanol was used, and it had a noticeable effect on both the qualitative and quantitative composition of the extracts. Extracts and preparations from *Ginkgo* are nowadays very popular, mainly in folk medicine. Medicinal effects of the extracts that are structured to free-radical scavenging and reducing power properties include: inhibition of lipid peroxidation, facilitation of the maintenance of the integrity and permeability of cell membranes, and protection of brain neurons against oxidative stress and post-ischemic injury induced by free radicals [30,31,32].

### 2.3. Total Polyphenol and Flavonoid Content

Polyphenols are known to be the main plant bioactive compounds with strong biological activity in the human body. These compounds can work as potential natural antioxidants in terms of their efficiency to act as both able radical scavengers and metal chelators [33]. Therefore, it was worthwhile to determine the total phenolic content in the *Ginkgo* leaf extracts. Total polyphenol content in the tested samples (Table 2) ranged from 1.476 to 2.804 mg GAE/g FM, with the highest content in the sample from the locality of Košice, followed by the sample from Rimavská Sobota. Similar to the results of antioxidant activity, a strong correlation between locality and the amount of polyphenolic compounds was observed. Free radical scavenging activity (DPPH) and total polyphenol content in evaluated samples showed a medium correlation (R^2^ = 0.740, *p* < 0.05). Total flavonoid content in evaluated samples (Table 2) ranged from 0.156 to 4.650 μg QE/g FM, with the best result in the sample from the locality of Košice, followed by the sample from the locality of Budimír. Reducing power and total flavonoid content in the evaluated samples showed a medium correlation (*R*^2^ = 0.757, *p* < 0.05), implying that the phenolic compounds were the major contributors to the observed antioxidant activity. Currently, according to Liu et al. [34], more than 34 flavonoids have been isolated from *Ginkgo* leaves and more than 70 flavonoids have been tentatively identified by mass spectrometry. The major flavonol glycosides in *Ginkgo* are the derivatives of quercetin, kaempferol and isorhamnetin linked with glucose and rhamnose in different linkage forms and number. Kobus et al. [25] determined qualitatively the composition of flavonols in the aqueous acetonic, ethanolic, and water infusion extracts of green and yellow *Ginkgo* leaves. These authors found a significantly (*p* < 0.05) higher content of flavonol aglycones in the extracts from yellow *Ginkgo* leaves. The highest amounts of these components were determined in the aqueous acetonic extract from yellow leaves (4938 μg/g DM) and the water infusion extract from yellow leaves (3146 μg/g DM), while the lowest were detected in the water infusion extract from green leaves (878 μg/g DM) and the ethanolic extract from green leaves (512 μg/g DM). Regarding the extract preparations from yellow *Ginkgo* leaves, the dominant flavonol was myricetin accounting for 1683, 1116, and 680 μg/g DM in the yellow water, aqueous acetonic and ethanolic extracts, respectively. In the extracts from green *Ginkgo* leaves, kaempferol, myricetin, and isorhamnetin predominated in general and ranged from 153 to 661 μg/g DM extract. The quantitative differences in the flavonol aglycone profiles for the preparations likely stem from the changes that occur within *Ginkgo* trees during vegetative growth, primarily accumulation of polyphenols over the course of a longer growth period, which is characteristic of yellow leaves. In our study we tested ethanolic (80%) extracts from leaves. Ethanol is commonly used to extract tannins, polyphenols, and flavonols, and usually ethanolic extracts showed a noticeable antioxidant capacity. During water extraction, mainly anthocyanins and tannins leached into the polar solvent with only a small proportion of flavonols; hence, such preparations have lower antiradical-scavenging potentials [35]. Flavonoids are responsible for health-promoting properties due to their high antioxidant capacity both in vivo and in vitro systems. These compounds can raise human protective enzyme systems. Several clinical studies have confirmed protective effects of flavonoids against many infectious (bacterial and viral) and civilization diseases, such as cardiovascular and digestive disorders, cancers, and other age-related health problems [36]. Being plant phytochemicals, flavonoids cannot be synthesized in humans and animals; they are essential and must be delivered daily to the human body. Dietary intake of flavonoids has been estimated from 100 to 1000 mg per day [37]. *Ginkgo* extracts and *Ginkgo*-related products can enrich human diet in flavonoids due to high content of ginkgolides and bilobalides—unique and specific flavonoid bioactive compounds identified only in *Ginkgo* [38].

### 2.4. Antimicrobial Activity with Disc Diffusion Method

The antimicrobial activity of leaf extracts of *G. biloba* varied with respect to the microorganisms and the locality of *G. biloba* used for extraction (Table 3 and Table 4). Disc diffusion method showed different antimicrobial effects of *G. biloba* against two different group of bacteria. Antimicrobial activity of *G. biloba* against *E. coli,* represented by inhibition zone diameter, ranged from 3.33 ± 1.53 mm (Šurianky) to 11.00 ± 1.00 mm (Košice), against *K. pneumoniae*—from 5.00 ± 1.00 mm (Šurianky) to 9.00 ± 1.00 mm (Košice), against *S. enteritidis*—from 4.67 ± 1.53 mm (Šurianky) to 12.33 ± 2.08 mm (Košice), against *S. sonei*—from 4.00 ± 1.00 mm (Palárikovo) to 12.33 ± 1.53 mm (Košice), and against *Y. enterocolitica*—from 4.00 ± 1.00 mm (Lučenec) to 11.00 ± 1.00 mm (Košice).

In the study of Sati et al. [39], it was reported that the diameter of growth inhibition zones of Gram negative bacteria was between 2.33–6.17, 2.27–4.83, and 1.83–4.67 mm, when methanol, ethyl acetate, and *n*-butanol extracts were tested (at concentration 40 mg/0.1 mL), respectively. In our study, ethanol extract was shown to have the greatest antimicrobial effect on bacteria.

The results of antimicrobial activity tests of *G. biloba*, given as diameters of inhibition zones, against *B. thuringiensis* ranged from 2.00 ± 1.00 mm (Nová Ves nad Žitavou) to 8.00 ± 1.00 mm (Košice), against *C. perfringens*—from 3.67 ± 1.53 mm (Veľký Blh, Šurianky) to 8.00 ± 2.65 mm (Košice), against *H. influenzae*—from 2.67 ± 2.08 mm (Palárikovo) to 10.00 ± 2.00 mm (Košice), against *L. monocytogenes*—from 3.00 ± 1.00 mm (Nová Ves nad Žitavou) to 6.00 ± 4.58 mm (Košice) and against *S. aureus*—from 3.33 ± 1.53 mm (Topoľčianky) to 8.00 ± 1.00 mm (Košice). The inhibition zones of Gram-positive bacteria were 2.27–8.86, 2.23–7.66, and 2.13–5.37 mm for methanol, ethyl acetate, and n-butanol extracts according to Sati et al. [39], in case of Gram-negative bacteria, the inhibition diameters ranged between 2.33–6.17, 2.27–4.83 and 1.83–4.67 mm. Additionally, in our study, the results on inhibition of Gram-positive bacteria by ethanol extracts of *G. biloba* were similar to those previously reported [40]. Inhibition of Gram-positive bacteria and yeasts was reported by Mazzanti et al. [41] who studied the antimicrobial activity *G. biloba* leaf extracts. Leaf extracts of *G. biloba* were not efficient for inhibition of *B. subtilis* and *E. coli* in recent report of Fazal et al. [7]. The climatic conditions in different localities where *G. biloba* leaves were harvested may influence the inhibition test results. *G. biloba* leaves originating from a temperate climate may have increased antimicrobial activity against a wider range of microorganisms. *G. biloba* leaf extract has been found to exhibit antimicrobial properties against medically important pathogens, e.g., *Pneumocystis carinii* and *Listeria monocytogenes* [12,17]. Other parts of plants, not only the leaves, have also expressed the antimicrobial activity [42,43].

### 2.5. Minimal Inhibition Concentration (MIC)

Minimum inhibitory concentration of ethanol extracts of nine leaf samples of *Ginkgo biloba* L. towards Gram negative bacteria are summarized in Table 5. The values of minimal inhibition concentration MIC 50 and MIC 90 towards *E. coli* ranged from 85.3 and 99.6 µg/mL, respectively (sample Košice), to 263.3 and 285.9 µg/mL, respectively (samples: Rimavská Sobota, Budimír, Veľký Blh); MIC 50 and MIC 90 towards *K. pneumoniae* ranged from 85.3 and 99.6 µg/mL, respectively (sample Košice), to 263.3 and 285.9 µg/mL (samples: Rimavská Sobota, Topoľčianky); MIC 50 and MIC 90 towards *S. enterica* ranged from 85.3 and 99.6 µg/mL, respectively (sample Košice), to 263.3 and 285.9 µg/mL, respectively (samples: Topoľčianky, Nová Ves nad Žitavou, Šurianky); MIC 50 and MIC 90 towards *S. sonei* ranged from 128.1 and 136.4 µg/mL, respectively (sample Košice), to 263.3 and 285.9 µg/mL, respectively (samples: Rimavská Sobota, Budimír, Veľký Blh, Lučenec); MIC 50 and MIC 90 towards *Y. enterocolitica* ranged from 85.3 and 99.6 µg/mL, respectively (sample Košice), to 263.3 and 285.9 µg/mL, respectively (sample Topoľčianky, Nová Ves nad Žitavou). We found that all the samples showed similar antimicrobial activity when tested on Gram-negative bacteria. However, the sample from the locality of Košice showed more potential for further research and the strongest antimicrobial activity. Out of all the tested Gram-negative bacteria, *E. coli, K. pneumoinae* and *Y. enterocolitica* were found to be the most susceptible to the crude extracts of *Ginkgo biloba* leaves.

Minimum inhibitory concentration of ethanol extracts of nine leaf samples of *Ginkgo biloba* L. towards Gram-positive bacteria are summarized in Table 6. The values of MIC 50 and MIC 90 towards *B. thuringiensis* ranged from 85.3 and 99.6 µg/mL, respectively (sample Nová Ves nad Žitavou), to 263.3 and 285.9 µg/mL, respectively (samples: Rimavská Sobota, Budimír, Veľký Blh); MIC 50 and MIC 90 towards *C. perfringens* ranged from 128.1 and 136.4 µg/mL, respectively (samples: Rimavská Sobota, Košice), to 263.3 and 285.9 µg/mL, respectively (sample Šurianky); MIC 50 and MIC 90 towards *H. influenzae* ranged from 85.3 and 99.6 µg/mL, respectively (samples: Košice, Budimír), to 341.3 and 382.3 µg/mL, respectively (sample Topoľčianky); MIC 50 and MIC 90 towards *L. monocytogenes* ranged from 85.3 and 99.6 µg/mL, respectively (sample Košice), to 263.3 and 285.9 µg/mL, respectively (sample Šurianky); MIC 50 and MIC 90 towards *S. aureus* ranged from 64.2 and 72.2 µg/mL, respectively (sample Budimír), to 263.3 and 285.9 µg/mL, respectively (sample Šurianky). We found similar antimicrobial activity in all the samples when tested against Gram-positive bacteria. Although, again, the sample from Košice showed more potential for further research and the strongest antimicrobial activity. Out of all tested bacteria, *S. aureus* was found to be the most susceptible to *Ginkgo biloba* extracts compared to other bacteria.

Extracts of *Ginkgo biloba* obtained using methanol, hexane and dichloromethane were also evaluated in terms of inhibitory activity against five Gram-positive bacterial strains in a study by Tewari et al. [44]. The dichloromethane extract was the most effective against *E. coli, P. aeruginosa* and *P. fluorescens.* The values of MIC ranged from 3.51 to 8.19 mg/mL. Another report on the antimicrobial activity of *G. biloba* leaf extracts suggested that the methanol extract has the best activity [39]. In a report by Bajpai et al. [45], the results of screening for antibacterial activity showed that the essential oil form *G. biloba* leaves had a potential inhibitory effect on the cell viability of the tested pathogens.

Our study on the antimicrobial activity of *Ginkgo biloba* ethanol extracts showed results that contrast with other available reports. Mazzanti et al. [41] reported that the antimicrobial activity of three fractions of methanolic extracts of *Ginkgo biloba* leaves was effective towards Gram-positive bacteria only. Lee and Kim [46] found that *Ginkgo biloba* leaf-derived materials inhibited *Clostridium perfringens* and *Escherichia coli*, but did not inhibit anaerobic intestinal bacteria, such as *Bifidobacterium bifidum*, a common and beneficial lactic acid bacteria, or *Lactobacillus acidophilus*. Sati and Joshi [47] showed that the methanolic extract of *Ginkgo biloba* leaves inhibited *Escherichia coli, Bacillus subtilis* and some plant pathogenic bacterial strains. Tao et al. [40] reported antibacterial/antifungal activities and synergistic interactions between *Ginkgo biloba* polyprenols and eight compounds separated from *Ginkgo biloba* L. leaf lipids against *Salmonella enterica, Staphylococcus aureus* and *Aspergillus niger*.

### 2.6. MicroRNA-Marker Assay

Next-generation sequencing has allowed identification of 202(m)/201(f) known and 174(m)/174(f) novel miRNAs of male and female leaves of ginkgo that belong to 82 and 78 families [48]. In our study we used DNA markers for microRNA designed according to miRNA sequences of *Ginkgo biloba* identified in the above-mentioned report. They were sorted into three categories: highly conserved (miR160), moderately conserved (miR482) and novel types of miRNA (miR75) families. Three primer combinations were used to analyze nine genotypes of *Ginkgo biloba* grown in different areas of Slovakia. Three types of the miRNA-based PCR primers were also used by Wang et al. [48] in their study. The first type was designed based on the sequences of miR160, which is a member of a deeply conserved miRNA family in the ginkgo genome. Their evolutionary conserved character suggests their specific role in a variety of physiological processes in plants [48,49]. Moderately conserved miRNAs are presented by miR482 which is involved in defense mechanisms of the gingko genome. The species-specific miRNA5261 family may have a significant role in producing new functions in special species [48]. Finally, the novel miRNA type identified by high-throughput sequencing of the ginkgo genome is gbl-miR75, which has a role in specific regulation of defensive processes.

A total of 211 miRNA fragments were amplified in nine ginkgo genotypes by three miRNA-based molecular marker primer pairs. The number of bands produced per primer combination ranged from 51 (gb-miR5261_F/gb-miR5261_R), 60 (gb-miR482F/gb-miR482R) to 100 (gb-miR160_F/gb-miR75_R). The amplification properties of the miRNA-based markers are summarized in Table 7. It can be assumed that miRNA-based primers combined in the same stem-loop structure are able to produce fragments corresponding to precursor molecules of pre-miRNA. Fu et al. [22] suggested that miRNA-based primers may likely amplify regions between neighboring miRNAs.

**Figure 1:**
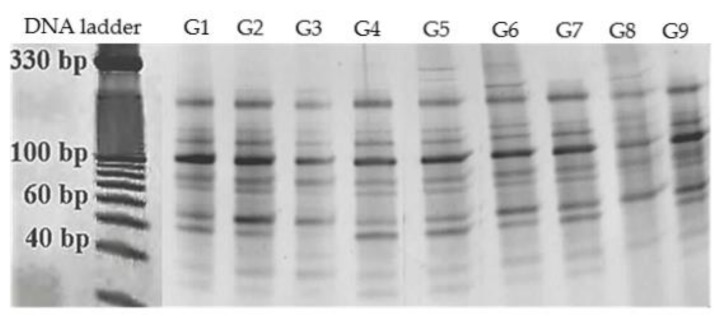
PCR amplification profile generated with miRNA-based primer pair gb-miR160F/gb-miR75R across 9 genotypes of *Ginkgo biloba*: G1—Nová Ves nad Žitavou, G2—Topoľčianky, G3—Lučenec, G4—Veľký Blh, G5—Rimavská Sobota, G6—Palárikovo, G7—Šurianky, G8—Košice and G9—Budimír.

**Figure 2:**
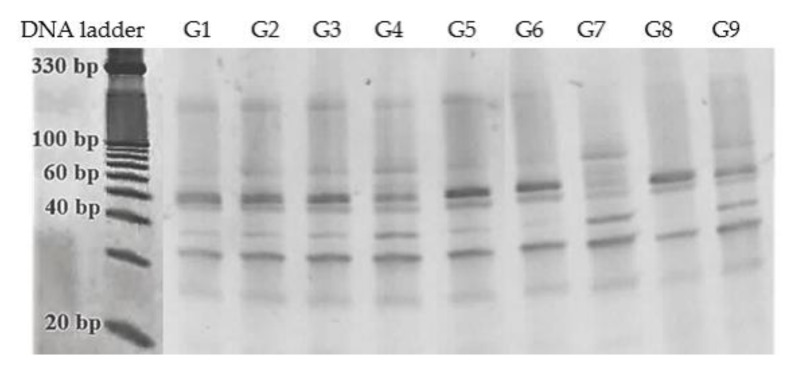
PCR amplification profile generated with miRNA-based primer pair gb-miR482F & gb-miR482R across 9 genotypes of *Ginkgo biloba*: G1—Nová Ves nad Žitavou, G2—Topoľčianky, G3—Lučenec, G4—Veľký Blh, G5—Rimavská Sobota, G6—Palárikovo, G7—Šurianky, G8—Košice and G9—Budimír.

Polymorphic information content was used to calculate allelic variation as the index of the individual primers’ practical applications. Table 7 presents the PIC value of each of the miRNA-based molecular marker. In general, PIC values of more than 0.5 indicate high polymorphism. The effect of the individual primers was measured by number of polymorphic, monomorphic and unique loci, percentage of polymorphism and PIC value were evaluated. These findings indicate that the molecular markers based on conservatively different types of microRNA sequences of *Ginkgo biloba* displayed different levels of polymorphism in all tested genotypes (Figure 1 and Figure 2). The possibility of a random combination of miRNA-based primers has the potential to generate more polymorphic loci. The sensitivity of the software system utilized for the evaluation fragments based on default parameters also allowed identification of unique miRNA loci. The unique loci were recorded in ginkgo genotypes that originated from Topoľčianky (2 unique loci), Rimavská Sobota (2 unique loci), Palárikovo and Budimír (one unique locus each).

Cluster analysis was performed for the data obtained from ginkgo trees from different localities in Slovakia to analyze whether these markers could be applied for detection of antioxidant and antimicrobial properties at the molecular level. Construction of a binary matrix for unweighted pair group method with arithmetic mean (UPGMA) analysis was based on the records of peak profiles from the SynGene GeneTools software. Each fragment was characterized in terms of the amount and volume of the peak profile in pixels. Based on these features it was possible to capture the unique loci and their specific position. Recorded profiles of miRNA fingerprints were analyzed under the default setting threshold in the analytical software in a manner in which all observable fragments were clearly identified

Hierarchical cluster trees (dendrograms) were constructed for each primer combination. The accuracy of the clustering based on our data was verified by cophenetic correlation coefficient. The values of the cophenetic correlation coefficients were as follows; 0.93 (cluster analysis by gb-miR160_F/gb-miR75 R, 0.95 (cluster analysis by gb-miR482_F/gb-miR482 R) and 0.98 (cluster analysis by gb-miR5261_F/gb-miR5261 R).

**Figure 3:**
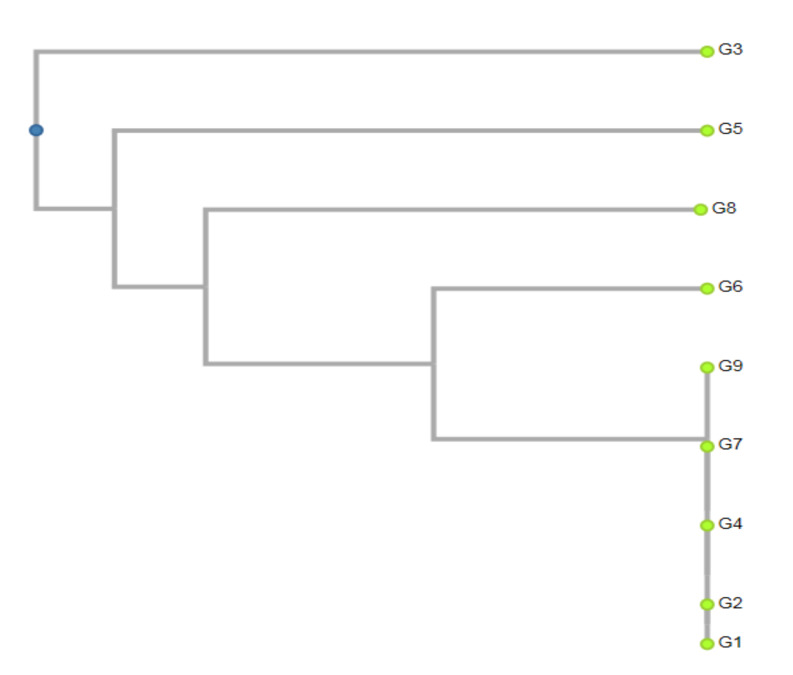
Dendrogram constructed by using the unweighted pair group method using arithmetic averages (UPGMA) cluster analysis of 9 genotypes of *Ginkgo biloba* on miRNA markers gb-miR160_F & gb-miR75_R. G1—Nová Ves nad Žitavou, G2—Topoľčianky, G3—Lučenec, G4—Veľký Blh, G5—Rimavská Sobota, G6—Palárikovo, G7—Šurianky, G8—Košice, G9—Budimír.

Application of different miRNA marker combinations resulted in the classification of nine ginkgo genotypes into one main cluster and all of the other samples were distinct from this cluster. Two genotypes from the western, one from the middle, one from the southern, and one from the eastern localities were clustered together into one cluster by the combination of deeply conserved gb-miR160 and novel type gb-miR75 of miRNA markers, (Figure 3). The remaining genotypes from the localities of Lučenec, Rimavská Sobota, Košice and Palárikovo were characterized by various levels of genetic diversity. It was the latter group of genotypes that showed significant antioxidant activity, total polyphenol and flavonoid content.

**Figure 4:**
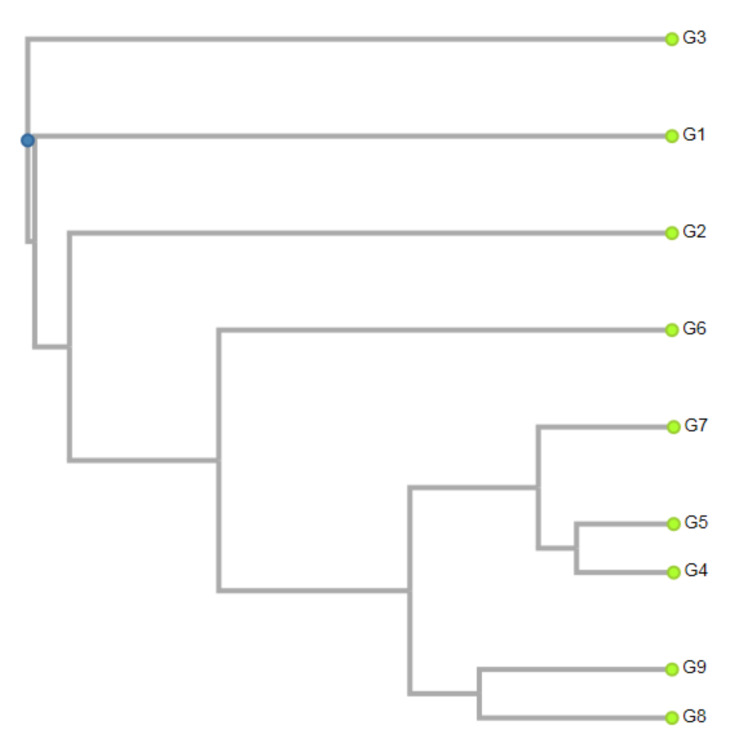
Dendrogram constructed by using unweighted pair group method using arithmetic averages (UPGMA) cluster analysis of 9 genotypes of *Ginkgo biloba* on miRNA markers gb-miR482_F/gb-miR482_R. G1—Nová Ves nad Žitavou, G2—Topoľčianky, G3—Lučenec, G4—Veľký Blh, G5—Rimavská Sobota, G6—Palárikovo, G7—Šurianky, G8—Košice, G9—Budimír.

The miRNA markers based on the moderately conserved microRNAs family allowed grouping of genotypes that reflected their locality. The trees from eastern (Košice and Budimír) and some from the southern (Veľký Blh and Rimavská Sobota) parts of Slovakia were classified in two smaller sub-clusters, while the genotypes from the western (Topoľčianky, Nová Ves nad Žitavou and Lučenec) and some from the southern (Šurianky and Palárikovo) localities were classified individually one by one according to their habitats (Figure 4). The ginkgo trees from Lučenec and Palárikovo belonged to separate clusters, as was also the case with clustering based on gb-miR160 and gb-miR75, which corresponds to their outstanding properties.

**Figure 5:**
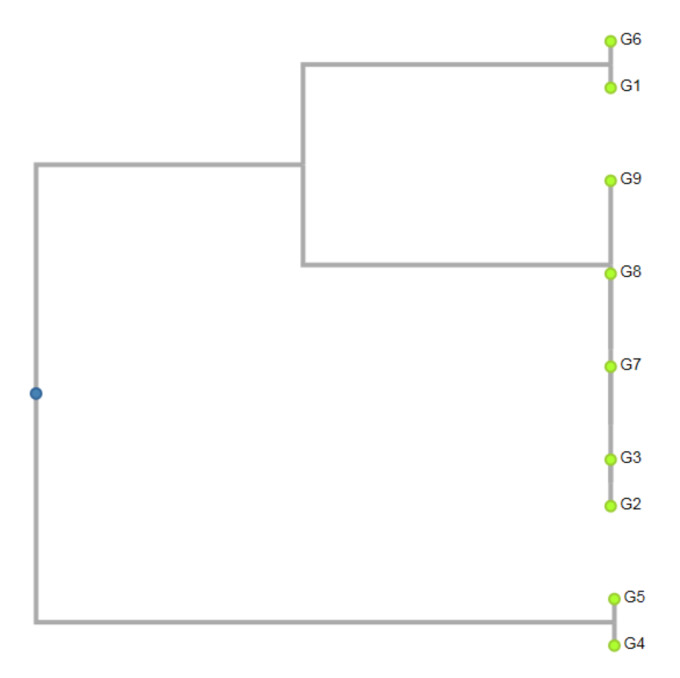
Dendrogram constructed by using unweighted pair group method using arithmetic averages (UPGMA) cluster analysis of 9 genotypes of *Ginkgo biloba* on miRNA markers gb-miR5261_F & gb-miR5261_R. G1—Nová Ves nad Žitavou, G2—Topoľčianky, G3—Lučenec, G4—Veľký Blh, G5—Rimavská Sobota, G6—Palárikovo, G7—Šurianky, G8—Košice, G9—Budimír.

Interestingly, based on the species-specific gb-miR5261 markers, almost all the genotypes of ginkgo were divided into three groups that corresponded to the location of growth (Figure 5). The major group consisted of genotypes that originated from the western (Topoľčianky and Lučenec), eastern (Košice and Budimír) and southern (Šurianky) localities. The second group consisted of trees from the western (Nová Ves nad Žitavou) and southern (Palárikov) localities, one from each. Finally, in the third group, trees from the southern (Veľký Blh and Rimavská Sobota) localities were grouped. The ginkgo genotypes that originated from the southern and western localities failed to cluster within one group.

The gingko leaves from Košice, Budimír, Lučenec, Rimavská Sobota and Šurianky localities were outstanding in terms of antioxidant and antimicrobial properties. The highest total polyphenol and flavonoid content was recorded in the samples from Košice, Budimír and Rimavská Sobota. As for the molecular-marker assay analyses, some of the above-mentioned genotypes (from localities of Lučenec, Rimavská Sobota and Košice) were excluded as individual exemplars, in particular by the analyses of the combination of the deeply conserved and novel type of miRNA markers (gb-miR160 & gb-miR75) as well as the moderately conserved markers (gb-miR482).

Predicted targets of miRNA160 include auxin response factor (ARF). This factor binds to promoters of auxin response elements and was reported to have a role in plant responses to diverse environmental factors [50]. The predicted targets of miRNA482 and gbl-miR75 represent genes encoding disease resistance proteins (disease resistance protein RPM1; disease resistance protein RPS2; DNA-directed RNA polymerases I, II, and III subunit RPABC1) [48]. This indicates that the defense mechanisms of *Ginkgo biloba* represent a major part of all biological processes in which the miRNAs play specific and essential roles. Our results demonstrate that the activity of these specific types of miRNA families was evenly intensive in all the analyzed genotypes. An interesting feature of primers based on miRNA sequences is that they can be combined randomly whereas SSR primers must be designed in fixed pairs. Hence, combined miRNA-based primers have the potential to generate more polymorphic fragments [22]. This was evidenced by our experimental data. The gb-miR160F/gbmiR75R primer combination produced about 55% more miRNA fragments in comparison to uncombined primer pair gb-miR482F/gbmiR482F. The miRNA markers are a dominant type of markers with putative functionality. The polymorphism observed when miRNA-based primers are used may be related to changes in regulation of expression of the target gene.

The applied type of molecular markers based on microRNAs is an appropriate tool for the characterization of individual genotypes of *Ginkgo biloba* originating from different localities in Slovakia. Moreover, it can be stated that miRNAs-based markers, targeted at the genetic determinants of adaptability and resistance mechanisms of *Ginkgo biloba,* could be used to select specific individuals with significant antimicrobial, antioxidant properties and morphological features.

## 3. Materials and Methods

### 3.1. Plant Materials and Sample Preparation

*Ginkgo biloba* L. leaves were collected from selected localities of Slovakia (Rimavská Sobota, Košice, Budimír, Veľký Blh, Palárikovo, Lučenec, Topoľčinanky, Nová Ves nad Žitavou, Šurianky) during the vegetation period of 2014 in the phase of maturity and stored at −50 °C until DNA extraction. The leaves were botanically identified at the Department of Genetics and Plant Breeding, Slovak University of Agriculture in Nitra. All the analyses were performed in triplicate. DNA extraction was performed form the pooled leaf material for each of the evaluated trees. All chemicals were analytical grade and were purchased from CentralChem (Bratislava, Slovakia), Penta (Prague, Czech Republic), Sigma–Aldrich (St. Louis, MO, USA) and Invitrogen (Camarillo, CA, USA).

The fresh leaves were used for preparation of ethanolic extracts. An amount of 1 g of each sample was extracted with 20 mL of 80% ethanol for 24 h. After centrifugation at 4000× *g* in Rotofix 32 A (Hettich Holding GmbH & Co. oHG, Kirchlengern, Germany) for 10 min, the supernatant was used for the measurements with DPPH and phosphomolybdenum methods, as well as for the analyses of total phenolic content and total flavonoid content.

For the antimicrobial activity and quantitative LC-MS/MS analysis the plant material was initially dried at room temperature in the dark. After drying, the material was crushed and weighed. The leaf powder was extracted with 96% ethanol p.a. (CentralChem, Bratislava, Slovakia) for 2 weeks, at a solid-to-liquid ratio of 1:10 (*w/v*), at room temperature, and in the dark. Exposure to sunlight was avoided in order to prevent the degradation of active compounds. Then, ethanol plant extracts were filtered through the Whatman No. 1 filter paper and dried using a rotary evaporator (Stuart RE300DB, Bibby Scientific Limited, Stone, UK) and vacuum pump (KNF N838.1.2KT.45.18, KNF Neuberger GmbH, Freiburg, Germany). Evaporation was performed under vacuum at - 40 °C in order to remove ethanol. For the antimicrobial assay, the crude plant extracts were dissolved in dimethyl sulfoxide (DMSO) (CentralChem, Bratislava, Slovakia) to 204.8 mg/100 mL as stock solution, which were stored at - 16 °C until use.

Total genomic DNA was extracted from leaves ground in liquid nitrogen according the protocol by Padmalantha and Prasad [51]. The extracted DNA was quantified using an Implen NanoPhotometer^®^, and diluted to 70 ng/μL.

### 3.2. LC-MS/MS Quantitative Analysis

For quantitative examination, an Agilent 1200 series liquid chromatograph coupled to 6410B triple-quadrupole mass spectrometer with electrospray ionization (ESI) was used (Agilent Technologies, Palo Alto, CA, USA). MassHunter ver. B.03.01. software was used for instrument control and data analysis. Analytical column used for separation was a Syncronis C18-column (100 × 2.1 mm, 1.7 μm particle size, ThermoFisher Scientific). The mobile phase consisted of (A) ultrapure water with 1% formic acid and (B) acetonitrile (MS grade). The injection volume for the sample was 10 μL, elution gradient program was 5–95% B for 20 min, with the flow rate of 0.3 mL/min. The injector volume for sample and standard compounds was 5 μL. The eluate was forwarded, without flow splitting, into an ESI ion source with following settings: drying gas (N2) temperature, 350 ℃; flow, 9 L/min; nebulizer gas pressure, 40 psi; capillary voltage, 4 kV, negative polarity. All compounds were quantified in dynamic MRM mode (multiple reaction monitoring mode) [52]. All the standards were dissolved in methanol to prepare stock solutions of 10 mg/mL. The mix of stock solutions was prepared, with concentration of each compound being 100 μg/mL. The mix was subsequently serially diluted, giving working standard solutions with concentrations ranging from 25.0 × 10^3^ ng/mL to 1.53 ng/mL. Calibration curves were based on these solutions. Concentrations of investigated compounds in extracts were determined on the basis of peak areas by using the equation of linear regression obtained from the calibration data (*R*^2^ = 0.995). The optimized parameters and target ions are presented in Table 8.

### 3.3. Analyses of Antioxidant Activity

#### 3.3.1. Free Radical Scavenging Activity

A 2,2-diphenyl-1-picrylhydrazyl (DPPH) was used to determine of free radical scavenging activity in accordance with the method describe by Sánchez-Moreno et al. [53]. Briefly, the absorbance of samples (0.4 mL) and alcohol solution DPPH (3.4 mL) was measured at a wavelength of 515 nm using a spectrophotometer (6405 UV/Vis, Jenway, Stone, England). The results of antioxidant activity were expressed as Trolox (6-hydroxy-2,5,7,8-tetramethylchroman-2-carboxylic acid) equivalent in mg/g.

#### 3.3.2. Molybdenum-Reducing Antioxidant Power

The method described by Prieto et al. [54] was used to determine molybdenum-reducing antioxidant power. Briefly, the sample (1 mL) was mixed with monopotassium phosphate (2.8 mL, 0.1 M), sulfuric acid (6 mL, 1 M), ammonium heptamolybdate (0.4 mL, 0.1 M) and distilled water (0.8 mL). Then, the mixture prepared in this way was incubated at 90 °C for 120 min and rapidly cooled. The absorbance at 700 nm was measured using above mentioned spectrophotometer and the results were presented as Trolox equivalent in mg/g.

#### 3.3.3. Total Polyphenol Content

A standard Singleton’s et al. [55] method was used to measure the total polyphenol content in analyzed samples. The results of absorbance at 700 nm measured spectrophotometrically (6405 UV/Vis, Jenway, Stone, England) were then calculated and expressed as gallic acid equivalents expressed in mg/g.

#### 3.3.4. Total Flavonoid Content

Total flavonoid content was measured spectrophotometrically (6405 UV/Vis, Jenway, Stone, England) at 415 nm accordingly to Willett’s [56] method. Briefly, to 0.5 mL of sample, ethanolic solution of aluminum chloride (10% w/v), potassium acetate (0.1 mL of 1 M) and distilled water (4.3 mL) were added. Then, the mixture was incubated in the dark for 30 min. Obtained results were expressed in mg/g quercetin equivalents.

### 3.4. Detection of Antimicrobial Activity

Altogether ten strains of microorganisms were tested in the study, including five Gram-negative bacteria (*Escherichia coli* CCM 3988, *Klebsiella pneumoniae* CCM 2318, *Salmonella enterica* subsp. *enterica* CCM 3807, *Shigella sonei* CCM 1373, *Yersinia enterocolitica* CCM 5671); and five Gram-positive bacteria (*Bacillus thuringiensis* CCM 19, *Clostridium perfringens* CCM 4991, *Haemophilus influenzae* CCM 4456, *Listeria monocytogenes* CCM 4699 and *Staphylococcus aureus* subsp. *aureus* CCM 2461). All tested strains were obtained from the Czech Collection of Microorganisms. The bacterial suspensions were cultured in nutrient broth (Imuna, Slovakia) at 37 °C for 24 h.

### 3.5. Disc Diffusion Method

A 0.1 mL of microorganisms suspension (10^6^ cfu/mL) was cultured on Mueller Hinton Agar (MHA, Oxoid, Basingstoke, United Kingdom). Filter paper discs (6 mm) were impregnated with 15 µL of plant extract and put on the inoculated agar. Cultivation was done at 4 °C for 2 h aerobically, with exception of *C. perfringens*, which was cultivated anaerobically, at 37 °C for 24 h. The diameters of the inhibition zones in millimeters were measured after incubation. All the tests were performed in triplicate.

### 3.6. Minimal Inhibitory Concentration

MICs were determined in Mueller Hinton broth (Biolife, Milan, Italy) by the microbroth dilution method. Dilutions were prepared according to the Clinical and Laboratory Standards Institute (CLSI, 2009) guidelines for bacteria. Plant extracts, dissolved in DMSO, were prepared to a final concentration of 204.8 mg/mL. Concentration of plant extracts were dissolved in DMSO for preparation of series of two-fold dilutions for final concentration from 0.33 to 682.67 μg/mL. Then 96-microwell plate was inoculated with microbial suspensions at a final density of 0.5 McFarland unit and incubated at 37 °C for 24 h. The microbial growth inhibition was measured by detecting absorbance at 517 nm (Biotek EL808, Biotek Instruments Inc., Winooski, VT, USA). Measurements were taken at the start and finish of the experiment. Differences between both measurements were evaluated as growth and values exceeding 715 nm from the mean absorbance were considered as error. The positive control was microbial suspension without plant extracts but the negative control was pure DMSO. This experiment was repeated in eight replicates for higher accuracy.

### 3.7. miRNA-marker Assay

The primers for the miRNA-marker assay were based on Wang et al. [48]. Sequences of following microRNA families were used: deeply conserved (gb-miR160), moderately conserved (gb-miR482), species-specific (gb-miR5261) and the novel miRNA family (gb-miR75) identified by high-throughput sequencing (Table 9). Mature miRNA sequences were used to design primers used in the study. The miRNA database was used for this purpose (http://www.mirbase.org/). Single forward and reverse primers were combined to perform a marker assay [57]. The following combinations of miRNA markers were used; gb-miR160_F/gb-miR75_R, gb-miR482_F/gb-miR482_R and gb-miR5261_F/gb-miR5261_R.

The marker assay was performed based on methodologies from Fu et al. [22] and Yadav et al. [20] which were modified for the purposes of the analysis [57,58]. PCR amplifications were performed in a 20-µL reaction mixture containing 70 ng of genomic DNA, 10 pmol.dm-3 of each primer, 2 units of DreamTaq DNA polymerase, 0.8 mmol.dm-3 dNTPs (Bioline) and 1×DreamTaq Buffer (KCl, (NH_4_)_2_SO_4_, 20 mmol/dm MgCl_2_). Touchdown PCR was used to amplify miRNA fingerprints with following parameters: 94 °C for 5 min; 5 cycles of 30 s at 94 °C, 45 s at 64 °C (1 °C decrease per cycle), and 60 s at 72 °C; 30 cycles of 30 s at 94 °C, 45 s at 60 °C, and 60 s at 72 °C; final 72 °C for 10 min. The samples were subsequently stored at 8 °C. The PCR products were separated using 15% TBE-Urea polyacrylamide gels, running in 1×TBE Running Buffer at a constant power of 90 V, 25 mA for 120 min. A 10-bp DNA ladder (Invitrogen) was used for size determination. The polyacrylamide gels were stained with the GelRedTM Nucleic Acid Gel stain and visualized in the G-Box Syngene electrophoresis documentation system. For the recording of loci number and identification of unique fragments, the gels were analyzed by the GeneTools software, GeneSnap version 7.09.17 (Syngene).

### 3.8. Statistical Analysis

For the statistical analyses of antioxidant activity data, the SAS program was used (The SAS system v9.2.). Correlation coefficients were calculated with CORR analysis [59]. For antimicrobial activity, the coefficients of variation and frequency of size of inhibition zones were calculated with Statgraphic (Statpoint Technologies, Warrenton, VA, USA). Absorbance values, before and after the incubation, determined during MIC analysis were used as a set of binary values to assign the exact concentrations. Probit analysis in STATGRAPHIC Centurion XVI software was used.

The bands for each miRNA-based allele were scored in terms of their presence (1) or absence (0). Scored data were used for the estimation of Jaccard´s similarity coefficient and this similarity matrix was used in cluster analysis using the unweighted pair group method using arithmetic averages (UPGMA) according to Garcia-Vallve et al. [60]. Polymorphism information content (PIC) value was calculated based on Fu et al. [22].

## 4. Conclusions

*Gingko biloba* is a plant known for its pronounced biological activity, yet its specific properties are dependent on the place of origin and agro-ecological conditions. The aim of this study was to characterize plants obtained from different Slovakian localities and leaf extracts obtained from them. Among the analyzed *Ginkgo* extracts, the sample from the locality of Košice was the highest in antioxidant activity as well as having the richest polyphenol and flavonoid content. Moreover, the obtained data indicates that *Ginkgo biloba* possess a remarkably high inhibitory activity against a wide spectrum of Gram-positive and Gram-negative bacteria. Molecular characterization of *Gingko* genotypes with the use of miRNA-based markers, made possible by sufficient polymorphism and stability, allowed determination of the genetic relationships between them and revealed regional similarities. Moreover, among specimens that showed outstanding properties in terms of biological activity, three were classified as individual exemplars using the UPGMA method and the results of the analyses of the combination of deeply conserved and novel types of miRNA markers as well as moderately conserved markers.

## Figures and Tables

**Table 1:** The content (μg/g) of 15 phenolic compounds in the nine extract samples.

No.	t*_R_*	Compound	Concentration (μg/g)								
			S1	S2	S3	S4	S5	S6	S7	S8	S9
1	3.49	Syringic acid	149.23 ± 0.78	189.38 ± 0.98	49.33 ± 0.26	77.68 ± 0.54	38.23 ± 0.33	133.58 ± 0.89	154.56 ± 0.78	197.36 ± 1.23	111.09 ± 1.16
2	3.68	Protocatechuic acid	298.23 ± 1.69	111.23 ± 1.22	147.66 ± 1.98	133.28 ± 0.23	122.56 ± 0.28	97.58 ± 0.79	277.38 ± 2.03	354.68 ± 3.05	322.21 ± 3.66
3	4.69	Chlorogenic acid	168.65 ± 1.65	223.35 ± 1.98	109.88 ± 1.11	54.33 ± 0.12	57.69 ± 0.68	244.36 ± 2.68	111.56 ± 1.86	97.33 ± 1.99	113.05 ± 1.53
4	5.52	Caffeic acid	211.87 ± 2.02	165.35 ± 1.63	247.21 ± 2.68	111.28 ± 1.12	120.33 ± 1.56	168.69 ± 1.58	285.69 ± 2.78	77.89 ± 1.27	135.42 ± 2.01
5	5.72	Quercetin-3-*O*-rutinoside	366.11 ± 2.26	498.38 ± 2.89	287.23 ± 2.21	228.36 ± 1.99	211.23 ± 1.46	387.77 ± 4.39	311.12 ± 3.21	226.25 ± 2.60	289.99 ± 2.25
6	6.38	*p*-Coumaric acid	186.22 ± 0.58	97.35 ± 0.59	101.36 ± 1.25	198.88 ± 1.78	91.13 ± 1.43	94.45 ± 1.25	177.58 ± 1.36	197.23 ± 2.68	177.07 ± 1.63
7	6.47	Quercetin-3-*O*-glucoside	111.59 ± 1.19	199.88 ± 0.77	144.58 ± 1.36	77.21 ± 0.38	222.34 ± 3.01	146.55 ± 0.88	77.58 ± 1.25	56.28 ± 0.34	98.22 ± 0.47
8	6.57	Kaempferol-3-*O*-glucoside	44.36 ± 0.21	31.44 ± 0.23	35.88 ± 0.15	27.12 ± 0.18	14.23 ± 0.07	24.55 ± 0.79	39.88 ± 0.13	37.69 ± 0.45	29.28 ± 0.04
9	6.75	Isorhamnetin-*O*-glucoside	3.56 ± 0.09	7.58 ± 0.08	6.25 ± 0.04	7.21 ± 0.11	1.28 ± 0.03	4.54 ± 0.02	9.11 ± 0.04	3.11 ± 0.08	4.66 ± 0.11
10	6.93	Luteolin-7-*O*-glucoside	9.28 ± 0.12	14.46 ± 0.09	7.36 ± 0.08	1.54 ± 0.05	2.56 ± 0.02	16.68 ± 0.08	17.14 ± 0.05	7.55 ± 0.03	11.69 ± 0.13
11	7.09	Rosmarinic acid	59.26 ± 0.23	116.35 ± 1.11	99.89 ± 1.22	12.98 ± 0.03	7.59 ± 0.02	77.68 ± 0.36	44.39 ± 0.38	69.99 ± 0.37	49.99 ± 0.45
12	7.34	Isorhamnetin	11.29 ± 0.08	29.23 ± 0.16	7.59 ± 0.03	3.23 ± 0.05	4.26 ±0.03	19.66 ± 0.12	24.29 ± 0.11	2.68 ± 0.04	36.55 ± 0.22
13	8.46	Luteolin	41.26 ± 0.15	71.77 ± 1.69	11.23 ± 0.05	19.28 ± 0.14	17.68 ± 0.08	31.11 ± 0.15	51.36 ± 0.69	111.23 ± 0.77	41.02 ± 0.28
14	8.52	Quercetin	68.25 ± 0.14	223.99 ± 2.98	211.39 ± 1.29	122.44 ± 2.29	158.68 ± 1.57	199.26 ± 1.28	58.11 ± 0.11	114.44 ± 0.46	77.68 ± 0.43
15	9.50	Kaempferol	17.52 ± 0.13	84.22 ± 1.26	36.29 ± 0.12	29.56 ± 0.37	19.58 ± 0.08	56.47 ± 0.28	21.33 ± 0.27	9.35 ± 0.11	21.65 ± 0.16

S1*—*Rimavská Sobota; S2*—*Košice; S3*—*Budimír; S4*—*Veľký Blh; S5*—*Palárikovo; S6*—*Lučenec; S7*—*Topoľčianky; S8- Nová Ves; S9*—*Šurianky. Results are given as a mean value of three measurements ± SD.

**Table 2:** Antioxidant activity, total polyphenol and flavonoid content of *Ginkgo* extract from selected Slovakian localities.

Sample/Locality	DPPH (mg TEAC/g FM)	MRP (mg TEAC/g FM)	TPC (mg GAE/g FM)	TFC (μg QE/g FM)
Rimavská Sobota	0.394 ± 0.011b	25.377 ± 0.086e	2.428 ± 0.046b	0.156 ± 0.015d
Košice	1.545 ± 0.112a	35.485 ± 0.464a	2.804 ± 0.131a	4.640 ± 0.025a
Budimír	1.224 ± 0.021a	26.226 ± 0.132d	1.967 ± 0.031d	2.982 ± 0.113a
Veľký Blh	0.502 ± 0.012b	26.306 ± 0.132d	1.695 ± 0.018e	1.193 ± 0.001bcd
Palárikovo	1.338 ± 0.021a	20.748 ± 0.418g	2.107 ± 0.100c	2.972 ± 0.045a
Lučenec	1.333 ± 0.051a	32.545 ± 0.177b	2.755 ± 0.073a	2.084 ± 0.057abc
Topoľčianky	0.558 ± 0.084b	26.266 ± 0.096d	1.785 ± 0.079e	0.676 ± 0.195d
Nová Ves	0.395 ± 0.022b	21.621 ± 0.298f	1.476 ± 0.083f	2.351 ± 0.059ab
Šurianky	0.997 ± 0.026b	26.835 ± 0.241c	2.095 ± 0.048cd	0.741 ± 0.015cd

Means ± standard deviations; TEAC – Trolox equivalent antioxidant capacity; MRP – molybdenum reducing power; FM – fresh matter; TPC – total polyphenol content; TFC – total flavonoid content; GAE – gallic acid equivalent; QE – quercetin equivalent; mean ± standard deviation; different letters in column indicate the mean values that were significantly different.

**Table 3:** Antimicrobial activity of *Gingko biloba* against Gram negative bacteria (in mm).

	Tested Gram-Negative Bacteria				
Sample Locality	*EC*	*KP*	*SE*	*SS*	*YE*
Rimavská Sobota	6.67 ± 1.53a	6.67 ± 1.53	6.67 ± 1.53a	5.33 ± 1.53a	6.33 ± 1.53a
Košice	11.00 ± 1.00ab	9.00 ± 1.00	12.33 ± 2.08ab	12.33 ± 1.53ab	11.00 ± 1.00ab
Budimír	6.00 ± 1.00b	7.33 ± 2.08	5.00 ± 1.00b	6.00 ± 1.00b	7.00 ± 2.65
Veľký Blh	8.00 ± 1.00c	5.67 ± 1.53	5.00 ± 2.00b	4.33 ± 0.58b	6.67 ± 1.53
Palárikovo	4.00 ± 1.00bc	5.00 ± 3.00	5.00 ± 1.00b	4.00 ± 1.00b	5.00 ± 1.00b
Lučenec	5.00 ± 1.00b	5.00 ± 1.00	6.67 ± 1.53b	7.00 ± 1.00b	4.00 ± 1.00b
Topoľčianky	6.00 ± 1.73b	6.67 ± 1.53	6.67 ± 1.53b	6.00 ± 2.65b	5.33 ± 1.15b
Nová Ves	5.67 ± 1.53b	5.33 ± 1.53	5.33 ± 0.58b	7.00 ± 1.00b	5.67 ± 0.58b
Šurianky	3.33 ± 1.53bc	5.00 ± 1.00	4.67 ± 1.53b	5.33 ± 0.58b	5.67 ± 2.08b

EC - *Escherichia coli* CCM 3988, KP - *Klebsiella pneumoniae* CCM 2318, SE - *Salmonella enterica* subsp. *enterica* CCM 3807, SS - *Shigella sonei* CCM 1373, YE - *Yersinia enterocolitica* CCM 5671; different letters in column indicate mean values that were significantly different.

**Table 4:** Antimicrobial activity of *Gingko biloba* against Gram positive bacteria (in mm).

	Tested Gram-Positive Bacteria				
Sample Locality	*BT*	*CP*	*HI*	*LM*	*SA*
Rimavská Sobota	5.00 ± 1.00	6.00 ± 1.00	6.67 ± 1.53	6.00 ± 1.00	5.33 ± 1.53
Košice	8.00 ± 1.00a	8.00 ± 2.65a	10.00 ± 2.00a	6.00 ± 4.58a	8.00 ± 1.00a
Budimír	3.00 ± 1.00a	5.00 ± 1.00	5.33 ± 1.53a	6.00 ± 1.00	6.00 ± 1.00
Veľký Blh	2.67 ± 1.53a	3.67 ± 1.53a	4.00 ± 1.00a	5.00 ± 1.00	4.67 ± 2.08
Palárikovo	3.00 ± 1.00a	4.00 ± 1.00	2.67 ± 2.08a	5.33 ± 1.53	5.00 ± 1.00
Lučenec	3.33 ± 2.08a	4.00 ± 1.00	3.00 ± 1.00a	3.67 ± 1.53	6.67 ± 1.53
Topoľčianky	4.33 ± 1.15a	6.00 ± 1.00	5.00 ± 1.00a	5.00 ± 1.00	3.33 ± 1.53a
Nová Ves	2.00 ± 1.00a	6.00 ± 1.00	3.00 ± 1.00a	3.00 ± 1.00a	4.00 ± 2.00
Šurianky	3.00 ± 1.00a	3.67 ± 1.53a	4.00 ± 1.00a	3.33 ± 1.53a	4.00 ± 1.00

BT—*Bacillus thuringiensis* CCM 19, CP—*Clostridium perfringens* CCM 4991, HI—*Haemophilus influenzae* CCM 4456, LM—*Listeria monocytogenes* CCM 4699, SA—*Staphylococcus aureus* subsp. *aureus* CCM 2461; letter “a” in column indicates the mean values that were significantly different.

**Table 5:** Minimal inhibition concentration of *Ginkgo biloba* L. (µg/mL) for Gram-negative bacteria.

		Tested Gram-Negative Bacteria				
Sample/Locality		*EC*	*KP*	*SE*	*SS*	*YE*
Rimavská Sobota	MIC 50	263.3	263.3	130.6	263.3	128.1
	MIC 90	285.9	285.9	143.8	285.9	136.4
Košice	MIC 50	85.3	85.3	128.1	128.1	85.3
	MIC 90	99.6	99.6	136.4	136.4	99.6
Budimír	MIC 50	263.3	170.7	130.6	263.3	128.1
	MIC 90	285.9	192.9	143.8	285.9	136.4
Veľký Blh	MIC 50	263.3	170.7	255.8	263.3	170.7
	MIC 90	285.9	192.9	272.0	285.9	190.8
Palárikovo	MIC 50	130.6	170.7	128.1	130.6	128.1
	MIC 90	143.8	192.9	136.4	143.8	136.4
Lučenec	MIC 50	263.3	130.6	128.1	263.3	128.1
	MIC 90	285.9	143.8	136.4	285.9	136.4
Topoľčianky	MIC 50	130.6	263.3	263.3	130.6	263.3
	MIC 90	143.8	285.9	285.9	143.8	285.9
Nová Ves	MIC 50	170.7	128.1	263.3	170.7	263.3
	MIC 90	192.9	136.4	285.9	192.9	285.9
Šurianky	MIC 50	130.6	128.1	263.3	130.6	128.1
	MIC 90	143.8	136.4	285.9	143.8	136.4

EC—Escherichia coli CCM 3988, KP—Klebsiella pneumoniae CCM 2318, SE—Salmonella enterica subsp. enterica CCM 3807, SS—Shigella sonei CCM 1373, YE—Yersinia enterocolitica CCM 5671.

**Table 6:** Minimal inhibition concentration of *Ginkgo biloba* L. (µg/mL) for Gram-positive bacteria.

		Tested Gram-Positive Bacteria				
Sample/Locality		*BT*	*CP*	*HI*	*LM*	*SA*
Rimavská Sobota	MIC 50	263.3	128.1	128.1	130.6	130.6
	MIC 90	285.9	136.4	136.4	143.8	143.8
Košice	MIC 50	170.7	128.1	85.3	85.3	85.3
	MIC 90	192.9	136.4	95.4	99.6	99.6
Budimír	MIC 50	263.3	128.1	85.3	130.6	64.2
	MIC 90	285.9	136.4	95.4	143.8	72.2
Veľký Blh	MIC 50	263.3	130.6	170.7	130.6	130.6
	MIC 90	285.9	143.8	192.9	143.8	143.8
Palárikovo	MIC 50	130.6	130.6	130.6	130.6	130.6
	MIC 90	143.8	143.8	143.8	143.8	143.8
Lučenec	MIC 50	263.3	170.7	263.3	128.1	128.1
	MIC 90	285.9	192.9	285.9	136.4	136.4
Topoľčianky	MIC 50	130.6	130.6	341.3	130.6	170.7
	MIC 90	143.8	143.8	382.3	143.8	192.9
Nová Ves	MIC 50	85.3	240.1	263.3	240.1	130.6
	MIC 90	99.6	389.9	285.9	389.9	143.8
Šurianky	MIC 50	130.6	263.3	263.3	263.3	263.3
	MIC 90	143.8	285.9	285.9	285.9	285.9

BT—Bacillus thuringiensis CCM 19, CP—Clostridium perfringens CCM 4991, HI—Haemophilus influenzae CCM 4456, LM—Listeria monocytogenes CCM 4699, SA—Staphylococcus aureus subsp. aureus CCM 2461.

**Table 7:** Details of miRNA marker assay with various parameters revealing the discriminatory power of each marker.

miRNA Marker	Number of Loci	Number of Polymorphic Loci	Number of Monomorphic Loci	Number of Unique Loci	Percentage of Polymorphism	PIC
gb-miR160F/gb-miR75R	14	3	9	2	21.43	0.91
gb-miR482F/gb-miR482R	14	9	1	4	64.29	0.90
gb-miR5261F/gb-miR5261R	8	3	5	0	37.5	0.83

PIC: polymorphic information content.

**Table 8:** LC-MS/MS parameters for standard phenolic compounds.

Compound	MS/MS Parameters				
	t*_R_*	Fragmentor Voltage (V)	Precursor Ion (m/z)	Product Ion (m/z)	Collision Energy (V)
Syringic acid	3.49	90	197	182	6
Protocatechuic acid	3.68	103	153	109	10
Chlorogenic acid	4.69	100	353	191	11
Caffeic acid	5.52	100	179	135	10
Quercetin-3-*O*-rutinoside	5.72	135	609	300	40
*p*-Coumaric acid	6.38	90	163	119	9
Quercetin-3-*O*-glucoside	6.47	210	463	300	35
Kaempferol-3-*O*-glucoside	6.57	200	447	284	30
Isorhamnetin-*O*-glucoside	6.75	210	477	315	25
Luteolin-7-*O*-glucoside	6.93	230	447	285	30
Rosmarinic acid	7.09	120	359	161	14
Isorhamnetin	7.34	155	315	300	23
Luteolin	8.46	135	285	133	30
Quercetin	8.52	130	301	151	19
Kaempferol	9.50	130	285	285	0

**Table 9:** Sequences of miRNA-based primers.

Primer	Sequences 5′- 3′
gb-miR160_F	TTAGTCTGCCTGGCTCCCTGTATG
gb-miR482_F	TGGGTTGTAGTCTTCAGGAGTGGG
gb-miR482_R	GAAGGCAATAGGAATGGGAGGATC
gb-miR5261_F	TTTGGAAAGTATTCGCATTGATTA
gb-miR5261_R	TATGGAACAAATTGCCACTCGGAT
gb-miR75_R	CCGGGCAGTAGGAATGGGAGGAAT
